# The Popular Treatment of Medical Subjects

**Published:** 1909-06-19

**Authors:** 


### The Hospital

A Journal of

The Medical Sciences and Hospital Administration.

New Series. No. 121, Vol. V. [No. 1193, Vol. XLVI.] SATURDAY, JUNE 19, 1909.

### THE POPULAR TREATMENT OF MEDICAL SUBJECTS.

There is a confirmed prejudice among medical men against the book which deals popularly with matters medical: a prejudice which is generic, impersonal, and may be looked for quite apart from the particular subject under notice or the man who has dealt with it. That such a fundamental bias should exist is not surprising, because the pseudo-medical brochure is a favourite resource of those who are anxious to exploit proprietary panaceas from purely commercial motives, and this business has reached so unconscionable a pitch that any one, no matter how irreproachable his professional standing, who appears to be dallying ever so remotely with public curiosity, stands in danger of sharing the condemnation of the advertising vendor of cure-alls. Nevertheless, the subject of admitting the public into our professional confidence is not one to be dogmatically dismissed, for there is much to be said on both sides, and the problem is worthy of consideration.

These reflections are prompted by a perusal of the latest addition to the new Library of Medicine, a book upon Drugs and the Drug Habit * from the pen of Dr. Harrington Sainsbury. The series to which the volume belongs is planned, we are told, upon the assumption that there are certain medical matters of the very gravest importance, which urgently claim the attention and appreciation not only of the medical man, but also of the intelligent layman. With this end in view the editor has concerned himself to select for his authors men of good professional standing, and for his subjects those matters of health which cannot be efficiently handled without the intelligent co-operation of the public.

Now every one will admit that these are laudable aims. There are many problems encountered by medical men, the satisfactory solution of which awaits the education of the public. Such, for example, are the prevention of consumption, the reduction of infantile mortality, and the bearing of heredity upon the race, all of which subjects are included in the series. In these matters the justification of the work lies upon the surface. It is common knowledge among the educated, and is becoming common knowledge even among the labouring class, that consumption is an infective malady propagated under well-ascertained conditions, that infantile mortality is for the most part preventable by the exercise of simple precautions, and that certain sins of fathers, and a good many of their physical disabilities, are liable to be visited upon their children. But no literature can be considered superfluous or to be deprecated which endeavours to drive home or disseminate these vital facts. Here it may be truly said that the prime function of the physician is an educative one, and that his success is to be measured by the efficiency of the education he imparts. But the mischief is that even in such cases as these, when the urgency of public co-operation with the profession is past dispute, one cannot but feel that the lessons inculcated in these volumes will not reach those who are most in need of them. For a man sufficiently intellectual to undertake the perusal of the New Library of Medicine has probably already gleaned the necessary knowledge in the course of his daily life. His infants will not die at the age of four months in consequence of being fed upon potato-skins and bacon or cheese, nor will his consumptive relatives remain an unrestricted menace to the health of their families. No. While one may applaud the purpose of such volumes and admit that their propriety is not debatable, it is hard to escape the conclusion that their practical value is likely to be disappointing. Unhappily, there seems to be no short cut to the education of those who most require it. To be achieved successfully it must be achieved laboriously, by oral demonstration and personally applied persuasion.

Turning now to the particular volume which forms the text of these remarks, one meets an entirely distinct phase of the question. How, it may be asked, is a disquisition upon the rationale of drug-treatment likely to benefit the layman? The answer is that a reasonable appreciation of the principles underlying the administration of drugs will tend to correct two extreme positions commonly assumed by different sections of the public. On the one hand, we have the confirmed sceptics who regard all medicaments as items of professional humbug; on the other, the confiding section so convinced of the virtues of the pharmacopœia that they are ready to expect miracles from it, and lose no opportunity of sampling its merits. If it were possible to persuade both these sections that there *is* a rationale underlying the administration of medicines, and that many of our drugs are potent and worthy servants (although the days of miracles are over), the relation of patient to physician would be decidedly smoothed, to the advantage of both. It will be seen that the claims of such a volume as this, considered purely on the score of its subject matter, stand on a lower plane than those of the other books mentioned above: on a lower plane, that is to say, of public utility, for in all other respects it is an excellent example of what a popular book on a medical subject should be. It is scholarly, thoughtful, pleasantly written, and altogether most readable. Although it deals with a variety of drug habits, there is nothing lurid or sensational in the treatment of the subject. It exhibits, in short, the good taste to be expected from the pen that wrote it. What, then, is there to grumble at? This. It is said that by a certain age every man is either a fool or a physician. The tag would be truer if it asserted that every man is a fool who thinks himself a physician, though he is, in fact, something else. For the superficial knowledge accumulated by a layman upon such a subject as medicine is peculiarly liable to engender distorted views, and to produce a being who, though never so apt at his own proper task, does harm both to himself and his intellectual reputation by the practice and preaching of his ill-digested learning.

It is hardly necessary to refer to the class of book which would seem to have for its motto "every man his own doctor," for we take it that there cannot be two opinions about the perniciousness of such publications. Every author who contributes to such a series as the New Library of Medicine must be conscious of the risks which await the task. Dr. Sainsbury has taken adequate pains to avoid them; but some of his fellows have, in our judgment, gone unwisely close to presenting a text-book of medicine for public perusal.

